# Supplementary material for: Inorganic Nanoparticles Applied for Active Targeted Photodynamic Therapy of Breast Cancer
Source: Pharmaceutics. 2021 Feb 24;13(3):296. doi: 10.3390/pharmaceutics13030296 (PMC7996317; doi:10.3390/pharmaceutics13030296)
Supplement: Supplementary file 1 [file pharmaceutics-13-00296-s001.pdf]

pharmaceutics

Supplementary Materials: Inorganic Nanoparticles Applied for Active Targeted Photodynamic Therapy of Breast Cancer

Hanieh Montaseri, Cherie Ann Kruger and Heidi Abrahamse

Citation: Montaseri, H.; Kruger, C.A.; Abrahamse, H. Inorganic Nanoparticles Applied for Active Targeted Photodynamic Therapy of Breast Cancer. *Pharmaceutics* **2021**, *13*, 296. https://doi.org/10.3390/pharmaceutics13030296

Publisher's Note: MDPI stays neutral with regard to jurisdictional claims in published maps and institutional affiliations.

Copyright: © 2021 by the authors. Submitted for possible open access publication under the terms and conditions of the Creative Commons Attribution (CC BY) license (http://creativecommons.org/licenses/by/4.0/).

Table S1. Spectroscopic and physicochemical properties of some photosensitizers utilized for PDT of cancer.

Type of PS	PS	Activation Wavelength (nm)	Solubility	LogP	Molar Extinction Coefficient (M^{-1}. cm^{-1})	Chemical Structure	Reference
BODIPY-type [1]	Boron dipyrromethene derivatives (BODIPY)	494–560	Hydrophilic	2.15–3.66	13,000–110,000	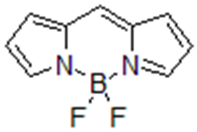	[1–4]
Bacteriophorbide derivatives	Palladium bacteriopheophorbide (TOOKAD)	763	Lipophilic	1.38	88,000	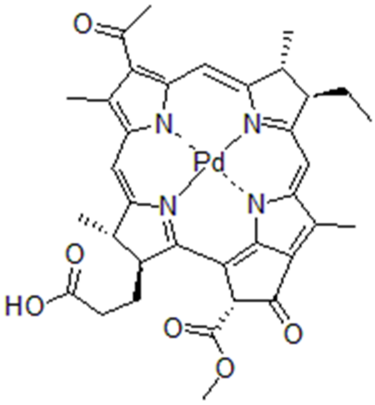	[5,6]
Chlorins	Chlorin e6 (Ce6)	670	Lipophilic	1.68	55,000	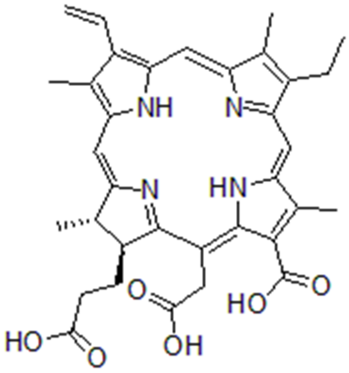	[7,8]
	mono-L-aspartyl chlorin e6 (NPe6)	664	Lipophilic	1.47	40,000	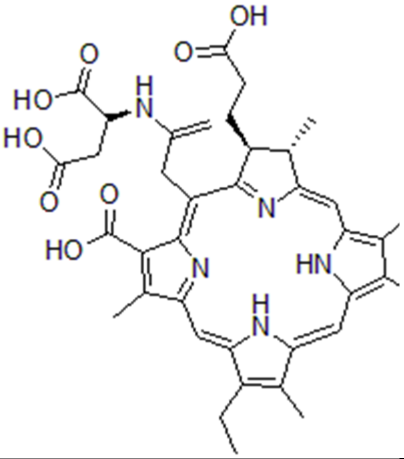	[5,9]

Temoporfin (Foscan®, mTHPC)	652	Lipophilic	7.40	30,000	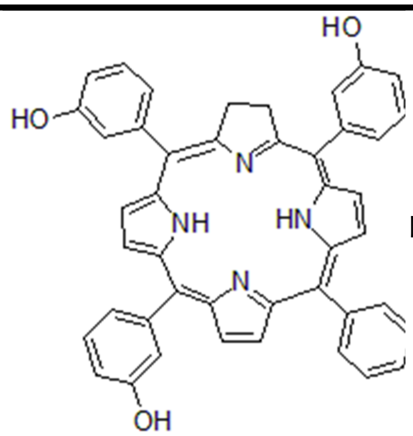	[10,11]
Purlytin (tin-ethyl-etiopurpurin)	664	Hydrophobic	Not identified	30,000	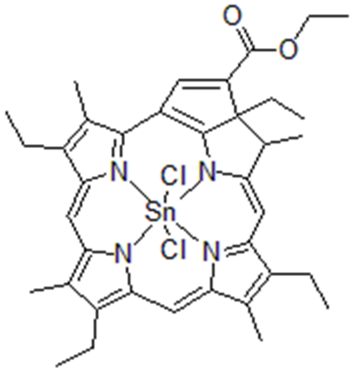	[5]
(2-[1-hexyloxyethyl]-2-devinyl pyropheophorbide-alpha (HPPH)	665	Hydrophobic	5.7	47,000	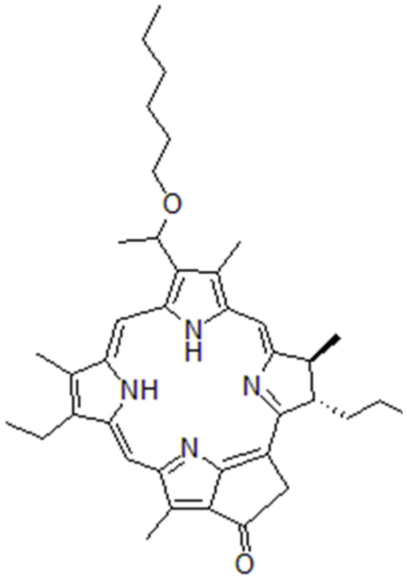	[5,12,13]

	Talaporfin sodium (LS11)	654	Hydrophilic	2.58	15,800	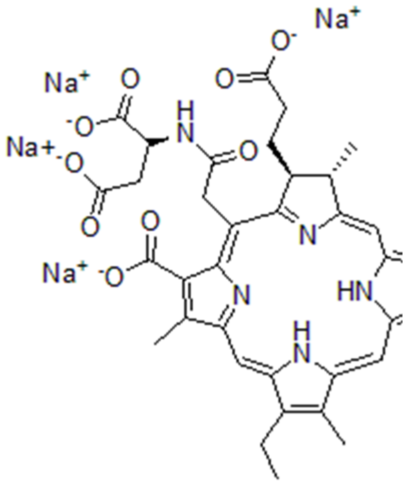	[14,15]
Chlorophyll derivatives	Pyropheophorbide-a (PPa)	669	Lipophilic	3.20	45,000	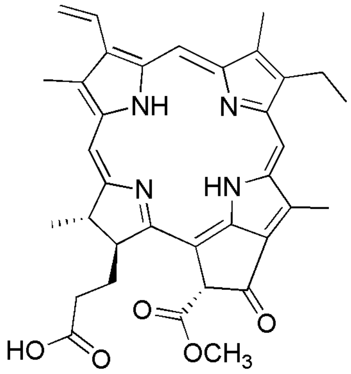	[16,17]
	IR-820 dye	819	Hydrophilic	Not identified	147,000	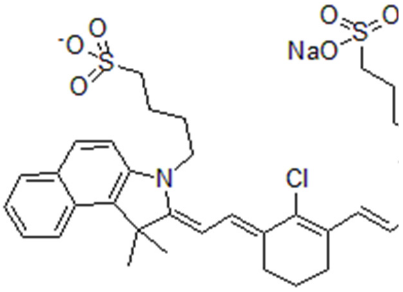	[18]
Cyanines	Merocyanin 540 (MC 540)	556	Lipophilic	3.90	110,000	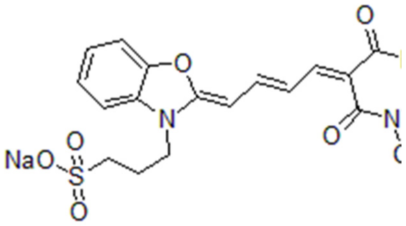	[5,19]
	Indocyanine green (ICG)	780	Hydrophilic	−0.29	343,000–57,3000	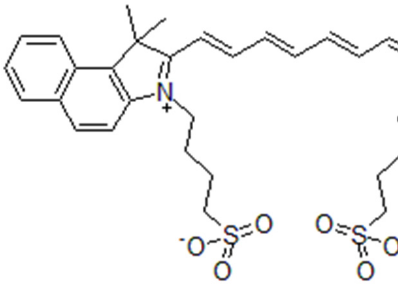	[20]

Dye-type	Methylene blue succinimidyl ester (MB-SE)	666	Hydrophilic	−0.785	82,000	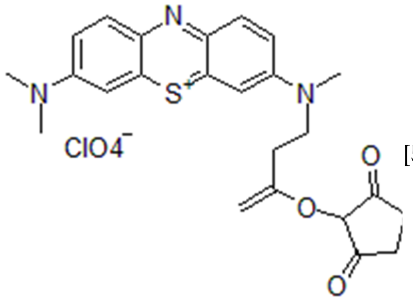	[5,9,21]
Hematoporphyrin derivative	Porfimer sodium	630	Lipophilic	3.96	3,000	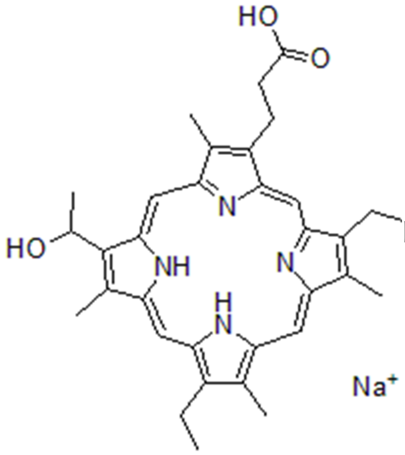	[5,9,11]
Herbal extracts	Pheophorbide A (PheoA)	667	Hydrophobic	Not identified	44,500	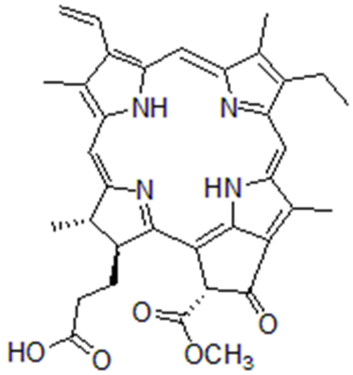	[22]
	Hypericin	590	Lipophilic	0.61	44,000	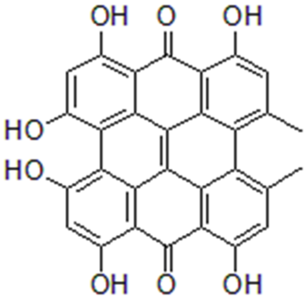	[5,23]
	Curcumin	428	Hydrophobic	3.0	55,000	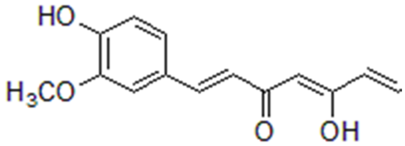	[5,24,25]

15(1)-Hydroxypurpurin-7-lactone ethyl methyl diester	668	Lipophilic	Not identified	40,400	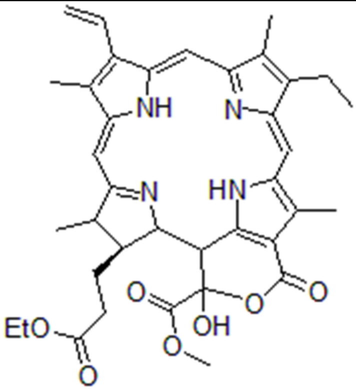	[26]
Tolyporphin	676	Hydrophilic	1.3	68,600	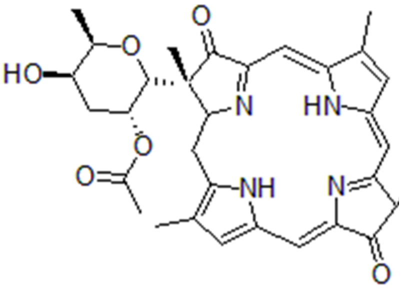	[27]
Aloe-emodin	430	Lipophilic	3.29	31,000	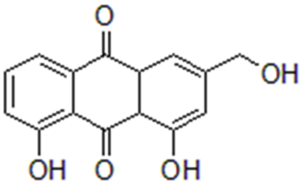	[28]
Chlorophyllin	650	Lipophilic	2.17	3,612	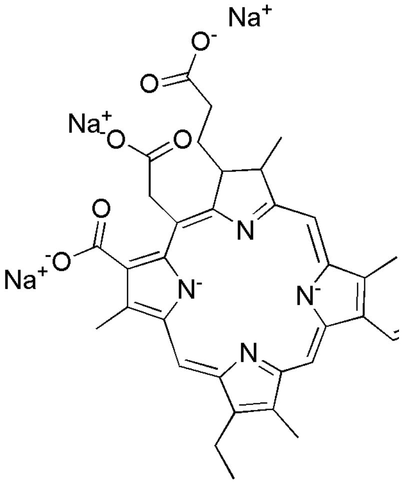	[29]
Hypocrellin A	580	Amphiphilic	41.6	12,000	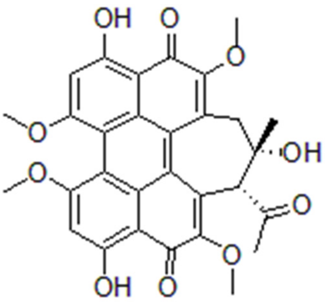	[30,31]

	Hypocrellin B	590	Amphiphilic	46.4	10,700	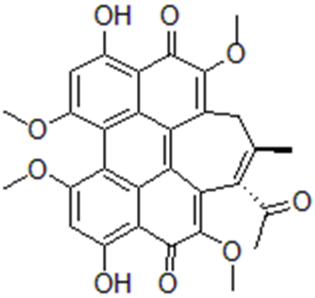	[30,31]
	Cercosporin	470	Lipophilic	4.92	23,600	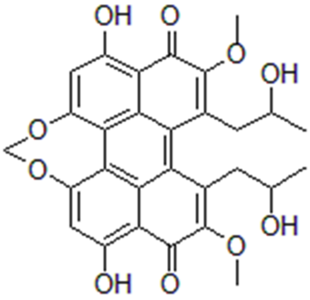	[32,33]
	Aluminium phthalocyanine (AlPc)	680	Lipophilic	8.91	126,000	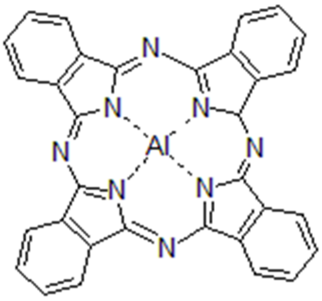	[11,34]
Metalophthalocyanines	Aluminum phthalocyanine tetrasulfonic acid (AlPcS4)	676	Hydrophilic	−0.74	158,000–200,000	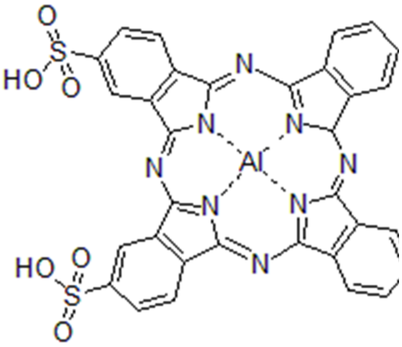	[5,11,34]
	Zinc phthalocyanine (ZnPc)	674	Lipophilic	8.50	274,000	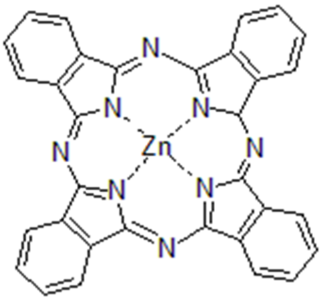	[11]

	Zinc phthalocyanine tetrasulfonic acid (ZnPcS4)	690	Amphiphilic	−1.00	295,000	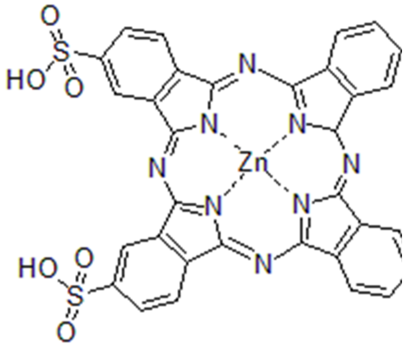	[11]
	Silicon phthalocyanine 4 (Pc4)	675	Lipophilic	Not identified	200,000	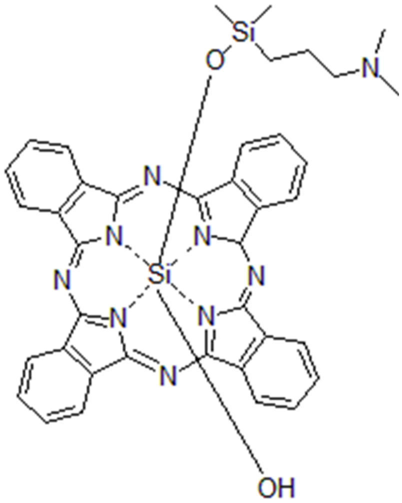	[5]
Naphthalocyanines	Silicon(IV) 2,3-naphthalocyanine bis(trihexylsilyloxide)	774	NA	Not identified	570,000	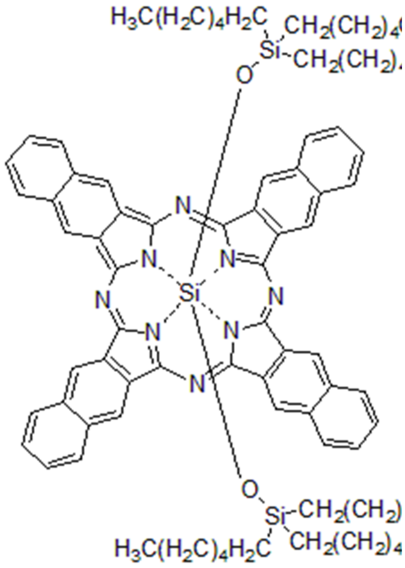	[35]
Phenothiazines	Toluidine blue	631	Lipophilic	0.31	51,000	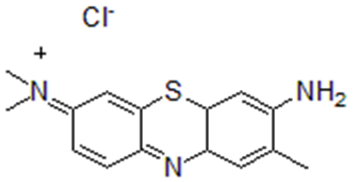	[5,36,37]

Porphyrins	Benzoporphyrin derivative monoacid (BPD)	690	Lipophilic	2.1	13,500	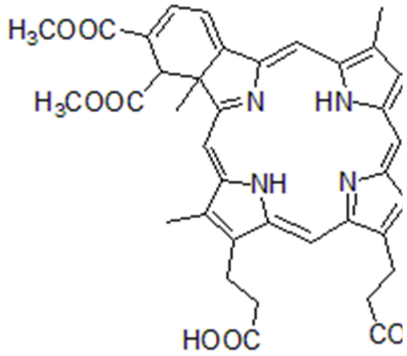	[38,39]
	5,10,15,20-tetrakis(4-hydroxyphenyl)-21H,23H-porphyrin (mTHPP)	420	Hydrophobic	Not identified	285,000	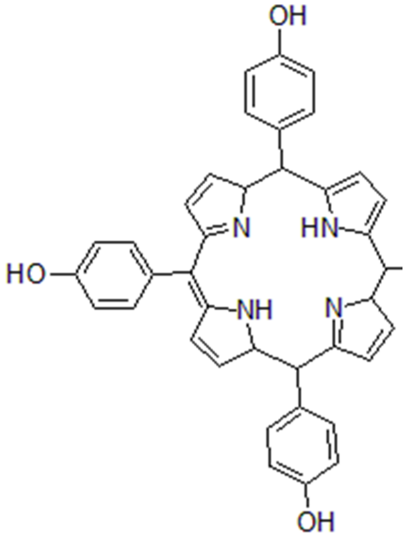	[40]
	Purpurin-18	699	Lipophilic	5.9	83,200 at 413 nm	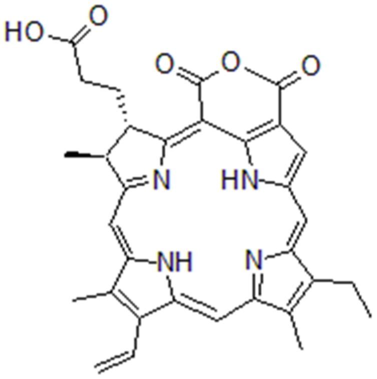	[41,42]
	Pyrolipid	665	Amphiphilic	Not identified	45,000	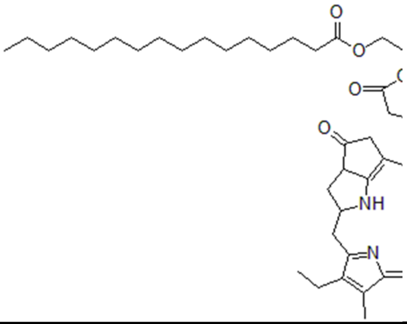	[43]

	Protoporphyrin IX (PpIX)	635	Hydrophilic	−1.89	275,000	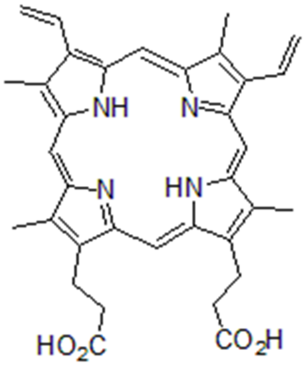	[44]
	hematoporphyrin derivative (HpD)	630	Lipophilic	3.25	3,000	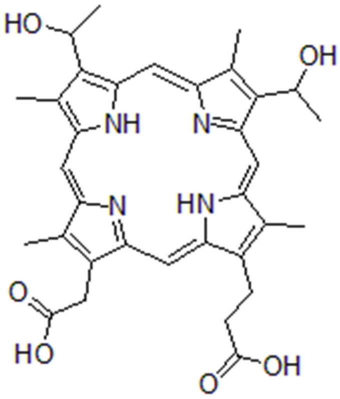	[11]
	Motexafin lutetium (Lu-Tex)	732	Hydrophilic	Not identified	42,000	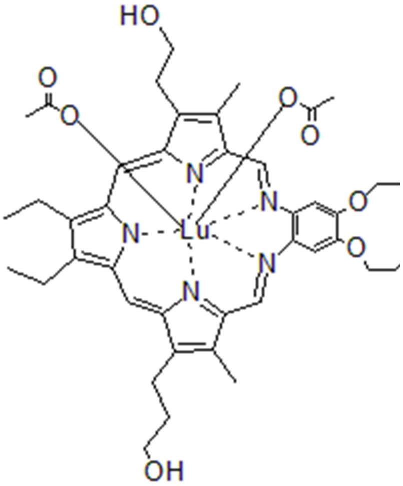	[5,45]
Xanthenes	Rose Bengal (RB)	549	Hydrophilic	3.46	100,000	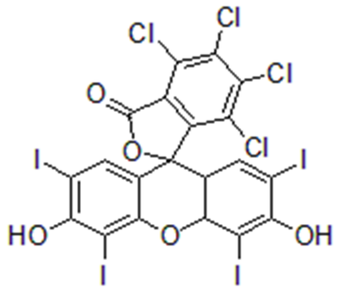	[5,9]

References

1. Lim, S.H.; Thivierge, C.; Nowak-Sliwinska, P.; Han, J.; Van Den Bergh, H.; Wagnieres, G.; Burgess, K.; Lee, H.B. In vitro and in vivo photocytotoxicity of boron dipyrromethene derivatives for photodynamic therapy. *J. Med. Chem.* **2010**, *53*, 2865–2874.
2. Romieu, A.; Massif, C.; Rihn, S.; Ulrich, G.; Ziessel, R.; Renard, P.-Y. The first comparative study of the ability of different hydrophilic groups to water-solubilise fluorescent BODIPY dyes. *New J. Chem.* **2013**, *37*, 1016–1027.
3. Kamkaew, A.; Lim, S.H.; Lee, H.B.; Kiew, L.V.; Chung, L.Y.; Burgess, K. BODIPY dyes in photodynamic therapy. *Chem. Soc. Rev.* **2013**, *42*, 77–88.

4. Kue, C.S.; Ng, S.Y.; Voon, S.H.; Kamkaew, A.; Chung, L.Y.; Kiew, L.V.; Lee, H.B. Recent strategies to improve boron dipyrromethene (BODIPY) for photodynamic cancer therapy: an updated review. *Photochem. Photobiol. Sci.* **2018**, *17*, 1691–1708.
5. Ormond, A.; Freeman, H. Dye sensitizers for photodynamic therapy. *Materials* **2013**, *6*, 817–840.
6. Brandis, A.; Mazor, O.; Neumark, E.; Rosenbach-Belkin, V.; Salomon, Y.; Scherz, A. Novel water-soluble bacteriochlorophyll derivatives for vascular-targeted photodynamic therapy: synthesis, solubility, phototoxicity and the effect of serum proteins¶. *Photochem. Photobiol.* **2005**, *81*, 983–992.
7. Bharathiraja, S.; Moorthy, M.S.; Manivasagan, P.; Seo, H.; Lee, K.D.; Oh, J. Chlorin e6 conjugated silica nanoparticles for targeted and effective photodynamic therapy. *Photodiagn. Photodyn. Ther.* **2017**, *19*, 212–220.
8. Isakau, H.; Parkhats, M.; Knyukshto, V.; Dzhagarov, B.; Petrov, E.; Petrov, P. Toward understanding the high PDT efficacy of chlorin e6–polyvinylpyrrolidone formulations: Photophysical and molecular aspects of photosensitizer–polymer interaction in vitro. *J. Photochem. Photobiol. B, Biol.* **2008**, *92*, 165–174.
9. Tanaka, M.; Kinoshita, M.; Yoshihara, Y.; Shinomiya, N.; Seki, S.; Nemoto, K.; Hirayama, T.; Dai, T.; Huang, L.; Hamblin, M.R. Optimal photosensitizers for photodynamic therapy of infections should kill bacteria but spare neutrophils. *Photochem. Photobiol.* **2012**, *88*, 227–232.
10. Biel, M. Advances in photodynamic therapy for the treatment of head and neck cancers. *Lasers Surg. Med.* **2006**, *38*, 349–355.
11. Weijer, R.; Broekgaarden, M.; Kos, M.; van Vught, R.; Rauws, E.A.; Breukink, E.; van Gulik, T.M.; Storm, G.; Heger, M. Enhancing photodynamic therapy of refractory solid cancers: Combining second-generation photosensitizers with multi-targeted liposomal delivery. *J. Photochem. Photobiol. C. Photochem. Rev.* **2015**, *23*, 103–131.
12. Yu, X.; Gao, D.; Gao, L.; Lai, J.; Zhang, C.; Zhao, Y.; Zhong, L.; Jia, B.; Wang, F.; Chen, X. Inhibiting metastasis and preventing tumor relapse by triggering host immunity with tumor-targeted photodynamic therapy using photosensitizer-loaded functional nanographenes. *ACS Nano* **2017**, *11*, 10147–10158.
13. Sengee, G.-I.; Badraa, N.; Lee, W.-k.; Shim, Y.-K. Photodynamic Effect of Water Soluble Piperazinium and Imidazolium Salts of HPPH on A549 Cancer Cells. *Bull. Korean Chem. Soc.* **2008**, *29*, 2505–2508.
14. Akimoto, J. Photodynamic therapy using talaporfin sodium and diode laser for newly diagnosed malignant gliomas. In *Clinical Management and Evolving Novel Therapeutic Strategies for Patients with Brain Tumors*, IntechOpen: London, UK, 2013.
15. Lustig, R.A.; Vogl, T.J.; Fromm, D.; Cuenca, R.; Alex Hsi, R.; D'Cruz, A.K.; Krajina, Z.; Turić, M.; Singhal, A.; Chen, J.C. A multicenter Phase I safety study of intratumoral photoactivation of talaporfin sodium in patients with refractory solid tumors. *Cancer* **2003**, *98*, 1767–1771.
16. Zheng, G.; Li, H.; Zhang, M.; Lund-Katz, S.; Chance, B.; Glickson, J.D. Low-density lipoprotein reconstituted by pyropheophorbide cholesteryl oleate as target-specific photosensitizer. *Bioconjugate Chem.* **2002**, *13*, 392–396.
17. Stamati, I.; Kuimova, M.K.; Lion, M.; Yahioglu, G.; Phillips, D.; Deonarain, M.P. Novel photosensitisers derived from pyropheophorbide-a: uptake by cells and photodynamic efficiency in vitro. *Photochem. Photobiol. Sci.* **2010**, *9*, 1033–1041.
18. Gao, F.; Bai, L.; Feng, X.; Tham, H.P.; Zhang, R.; Zhang, Y.; Liu, S.; Zhao, L.; Zheng, Y.; Zhao, Y. Remarkable In Vivo Nonlinear Photoacoustic Imaging Based on Near-Infrared Organic Dyes. *Small* **2016**, *12*, 5239–5244.
19. Toprak, M.; Aydın, B.M.; Arık, M.; Onganer, Y. Fluorescence quenching of fluorescein by Merocyanine 540 in liposomes. *J. Lumin.* **2011**, *131*, 2286–2289.
20. Yuan, B.; Chen, N.; Zhu, Q. Emission and absorption properties of indocyanine green in Intralipid solution. *J. Biomed. Opt.* **2004**, *9*, 497–504.
21. Hah, H.J.; Kim, G.; Lee, Y.E.K.; Orringer, D.A.; Sagher, O.; Philbert, M.A.; Kopelman, R. Methylene blue-conjugated hydrogel nanoparticles and tumor-cell targeted photodynamic therapy. *Macromol. Biosci.* **2011**, *11*, 90–99.
22. Eichwurzel, I.; Stiel, H.; Röder, B. Photophysical studies of the pheophorbide a dimer. *J. Photochem. Photobiol. B, Biol.* **2000**, *54*, 194–200.
23. Huygens, A.; Kamuhabwa, A.R.; Van Cleynenbreugel, B.; Van Poppel, H.; Roskams, T.; De Witte, P.A. In vivo accumulation of different hypericin ion pairs in the urothelium of the rat bladder. *BJU Int.* **2005**, *95*, 436–441.
24. Waranyoupalin, R.; Wongnawa, S.; Wongnawa, M.; Pakawatchai, C.; Panichayupakaranant, P.; Sherdshoopongse, P. Studies on complex formation between curcumin and Hg (II) ion by spectrophotometric method: A new approach to overcome peak overlap. *Cent. Eur. J. Chem.* **2009**, *7*, 388–394.
25. Priyadarsini, K. The chemistry of curcumin: from extraction to therapeutic agent. *Molecules* **2014**, *19*, 20091–20112.
26. Lim, S.H.; Lee, H.B.; Ho, A.S.H. A new naturally derived photosensitizer and its phototoxicity on head and neck cancer cells. *Photochem. Photobiol.* **2011**, *87*, 1152–1158.
27. Morlière, P.; Mazière, J.-C.; Santus, R.; Smith, C.D.; Prinsep, M.R.; Stobbe, C.C.; Fenning, M.C.; Golberg, J.L.; Chapman, J.D. Tolyporphin: a natural product from cyanobacteria with potent photosensitizing activity against tumor cells in vitro and in vivo. *Cancer Res.* **1998**, *58*, 3571–3578.
28. Zang, L.; Zhao, H.; Ji, X.; Cao, W.; Zhang, Z.; Meng, P. Photophysical properties, singlet oxygen generation efficiency and cytotoxic effects of aloe emodin as a blue light photosensitizer for photodynamic therapy in dermatological treatment. *Photochem. Photobiol. Sci.* **2017**, *16*, 1088–1094.
29. Uchoa, A.F.; Konopko, A.M.; Baptista, M.S. Chlorophyllin Derivatives as Photosensitizers: Synthesis and Photodynamic Properties. *J. Braz. Chem. Soc.* **2015**, *26*, 2615–2622.
30. He, Y.-Y.; Liu, H.-Y.; An, J.-Y.; Han, R.; Jiang, L.-J. Photodynamic action of hypocrellin dyes: structure–activity relationships. *Dyes Pigments* **1999**, *44*, 63–67.

31. Xie, J.; Ma, J.; Zhao, J. Prediction on amphiphilicity of hypocrellin derivatives. *Sci. China Ser. B.* **2002**, *45*, 251–256.
32. Mastrangelopoulou, M.; Grigalavicius, M.; Berg, K.; Ménard, M.; Theodossiou, T.A. Cytotoxic and Photocytotoxic Effects of Cercosporin on Human Tumor Cell Lines. *Photochem. Photobiol.* **2019**, *95*, 387–396.
33. Yamazaki, S.; Ogawa, T. The chemistry and stereochemistry of cercosporin. *Agric. Biol. Chem.* **1972**, *36*, 1707–1718.
34. Chan, W.-S.; Marshall, J.F.; Svensen, R.; Bedwell, J.; Hart, I.R. Effect of sulfonation on the cell and tissue distribution of the photosensitizer aluminum phthalocyanine. *Cancer Res.* **1990**, *50*, 4533–4538.
35. Duffy, M.J.; Planas, O.; Faust, A.; Vogl, T.; Hermann, S.; Schäfers, M.; Nonell, S.; Strassert, C.A. Towards optimized naphthalocyanines as sonochromes for photoacoustic imaging in vivo. *Photoacoustics* **2018**, *9*, 49–61.
36. Jebaramya, J.; Ilanchelian, M.; Prabahar, S. Spectral studies of toluidine blue o in the presence of sodium dodecyl sulfate. *Sensors* **2009**, *21*, 22.
37. Usacheva, M.N.; Teichert, M.C.; Biel, M.A. Comparison of the methylene blue and toluidine blue photobactericidal efficacy against gram-positive and gram-negative microorganisms. *Lasers Surg. Med.* **2001**, *29*, 165–173.
38. Jadia, R.; Kydd, J.; Rai, P. Remotely Phototriggered, Transferrin-Targeted Polymeric Nanoparticles for the Treatment of Breast Cancer. *Photochem. Photobiol.* **2018**, *94*, 765–774.
39. Aveline, B.M.; Hasan, T.; Redmond, R.W. The effects of aggregation, protein binding and cellular incorporation on the photophysical properties of benzoporphyrin derivative monoacid ring A (BPDMA). *J. Photochem. Photobiol. B, Biol.* **1995**, *30*, 161–169.
40. Narsireddy, A.; Vijayashree, K.; Irudayaraj, J.; Manorama, S.V.; Rao, N.M. Targeted in vivo photodynamic therapy with epidermal growth factor receptor-specific peptide linked nanoparticles. *Int. J. Pharm.* **2014**, *471*, 421–429.
41. Zhang, Y.; Zhang, H.; Wang, Z.; Jin, Y. pH-Sensitive graphene oxide conjugate purpurin-18 methyl ester photosensitizer nanocomplex in photodynamic therapy. *New J. Chem.* **2018**, *42*, 13272–13284.
42. Darmostuk, M.; Jurášek, M.; Lengyel, K.; Zelenka, J.; Rumlová, M.; Drašar, P.; Ruml, T. Conjugation of chlorins with spermine enhances phototoxicity to cancer cells in vitro. *J. Photochem. Photobiol. B, Biol.* **2017**, *168*, 175–184.
43. Ng, K.K.; Takada, M.; Jin, C.C.; Zheng, G. Self-sensing porphysomes for fluorescence-guided photothermal therapy. *Bioconjugate Chem.* **2015**, *26*, 345–351.
44. Xu, W.; Qian, J.; Hou, G.; Wang, Y.; Wang, J.; Sun, T.; Ji, L.; Suo, A.; Yao, Y. A dual-targeted hyaluronic acid-gold nanorod platform with triple-stimuli responsiveness for photodynamic/photothermal therapy of breast cancer. *Acta Biomater.* **2019**, *83*, 400–413.
45. Patel, H.; Mick, R.; Finlay, J.; Zhu, T.C.; Rickter, E.; Cengel, K.A.; Malkowicz, S.B.; Hahn, S.M.; Busch, T.M. Motexafin lutetium-photodynamic therapy of prostate cancer: short-and long-term effects on prostate-specific antigen. *Clin. Cancer Res.* **2008**, *14*, 4869–4876.
